# Evaluating the Effect of RNA Interference of Two Heat Shock Proteins (*HSPA1L*, *HSP90B1*) in Inducing Apoptosis in *Acyrthosiphon Pisum*

**Published:** 2026-01-06

**Authors:** Griffin Davies, James Balthazor

**Affiliations:** 1 Department of Biological Sciences, Fort Hays State University, Hays, United States of America; 2 Department of Chemistry, Fort Hays State University, Hays, United States of America

**Keywords:** RNAi, Pest mitigation, dsRNA, Knockdown, Silencing, Aphid, Unfolded protein response

## Abstract

*Acyrthosiphon pisum* (pea aphid) is a major pest of Fabaceae (legume) crops, causing direct feeding damage and transmitting plant diseases. Conventional control relies on broad-spectrum insecticides and natural enemies, both of which can harm non-target organisms and ecosystems. RNA interference (RNAi) offers a promising, species-specific alternative by silencing essential genes *via* double-stranded RNA (dsRNA). This study targeted two heat shock protein Genes-Heat Shock 70 kDa Protein 1-Like (*HSPA1L*) and Heat Shock Protein 90 kDa beta (Grp94) Member 1 (*HSP90B1*)-key players in protein folding, quality control, and stress response within the Unfolded Protein Response (*UPR*) pathway. These chaperones stabilize proteins, assist in folding newly synthesized polypeptides, and target misfolded proteins for degradation. Silencing these genes was hypothesized to cause accumulation of misfolded proteins, triggering Endoplasmic Reticulum (ER) stress, *UPR* overload, and ultimately apoptosis, leading to aphid mortality.

Total RNA was extracted from adult pea aphids, reverse-transcribed to cDNA, and used to synthesize gene-specific dsRNAs for *HSPA1L* and *HSP90B1*. These dsRNAs were encapsulated in Branched-Amphiphilic Peptide Capsules (BAPCs) and delivered orally at concentrations of 10 ng/μL and 100 ng/μL *via* artificial diet feeding. Survival was monitored every 6 hours for 48 hours on dsRNA diet, followed by transfer to healthy faba bean leaves. Preliminary studies indicated positive correlations between gene knockdown and elevated mortality. Results demonstrated significantly reduced survival in treated groups compared to controls at both concentrations (Kaplan-Meier survival analysis, log-rank p<0.001), supporting the induction of apoptosis. This approach highlights the potential of *UPR*-targeted RNAi for eco-friendly, targeted pest control.

## INTRODUCTION

The pea aphid, *Acyrthosiphon pisum*, is a phloem-feeding hemipteran pest that infests legumes worldwide, reducing crop yields through direct sap removal and vectoring viruses. Current management depends on chemical insecticides or biological control *via* predators (e.g., ladybeetles) and parasitoids, but these methods have drawbacks: Insecticides contribute to environmental contamination, non-target toxicity and resistance development, while biological control often provides inconsistent efficacy [1].

RNA interference (RNAi) provides a precise, sequence-specific gene silencing mechanism using double-stranded RNA (dsRNA), which triggers the cellular RNAi machinery to degrade target mRNA and prevent protein synthesis (NCBI). In aphids, oral and injection-based RNAi delivery has been successfully demonstrated for various genes, offering potential for species-specific pest management. This study focuses on genes in the Unfolded Protein Response (*UPR*), a conserved cellular pathway that maintains Endoplasmic Reticulum (ER) homeostasis by sensing misfolded proteins and activating adaptive responses, including chaperone upregulation, ER-Associated Degradation (ERAD) and if stress is unresolved apoptosis [2]. Pea aphids produce an unusually high number of unfolded proteins in their saliva, potentially rendering the *UPR* pathway particularly sensitive and making its components attractive targets for RNAi [3].

Previous research has established the feasibility of targeting *UPR* genes in *A. pisum* using RNAi. Ridder and investigated the effects of dsRNA-mediated knockdown of three key *UPR* genes: Activating Transcription Factor 4 (ATF4), Eukaryotic Translation Initiation Factor 2-Alpha Kinase (eIF2αK), and Inositol-Requiring Enzyme 1 (IRE1) [4]. Their feeding assays demonstrated that silencing these genes led to significant increases in aphid mortality, with positive correlations observed between gene knockdown efficiency and elevated death rates. Notably, some *UPR* genes produced stronger lethality than others, highlighting the pathway’s potential for pest control while underscoring the need to evaluate multiple components for optimal efficacy.

Building on this foundation, the current study targets two additional *UPR*-associated heat shock protein genes: *HSPA1L* (encoding a Hsp70-like protein) and *HSP90B1* (encoding Grp94, an ER-resident Hsp90 paralog). Heat shock proteins (HSPs) function as molecular chaperones that assist in protein folding, prevent aggregation, protect cells under stress, and contribute to ER quality control [5]. Hsp70 (*HSPA1L*) is involved in processes such as protein transport, proteasomal degradation and apoptosis regulation, while Grp94 (*HSP90B1*) specializes in calcium binding, client protein maturation and handling misfolded proteins in the ER [6,7]. Although these roles are well-characterized in mammals, they are conserved across eukaryotes, including insects.

Silencing *HSPA1L* was expected to disrupt apoptosis regulation and overall proteostasis, while *HSP90B1* knockdown should cause an irreparable buildup of misfolded proteins in the ER, overwhelming the *UPR* and triggering cell death. It was hypothesized that both genes would induce apoptosis and reduce aphid survival, contributing to the evaluation of *UPR* components as RNAi targets for sustainable pest management [8–10].

## MATERIALS AND METHODS

### RNA Isolation and cDNA Synthesis

Ten adult pea aphids were homogenized in 1 mL TRIzol reagent. After chloroform extraction and isopropanol precipitation, RNA purity (A260/A280 ≥ 1.90) was confirmed *via* nanodrop. cDNA was synthesized from 1.0 μg RNA using the iscript kit.

### dsRNA Synthesis and Visualization

Gene-specific dsRNAs were produced using a T7 RNA polymerase kit with *HSPA1L* and *HSP90B1*-specific primers and verified by agarose gel electrophoresis.

### Diet Preparation and Feeding Assay

dsRNAs were encapsulated in BAPCs (200 μM) and diluted with Akey-Beck diet to 10 ng/μL and 100 ng/μL. Fifty adult aphids per group (5 groups total: Control+4 treatments) were fed dsRNA diet *via* parafilm for 48 h, then transferred to faba leaves. Survival was recorded every 6 h until complete mortality.

## RESULTS

**Figure 1:**
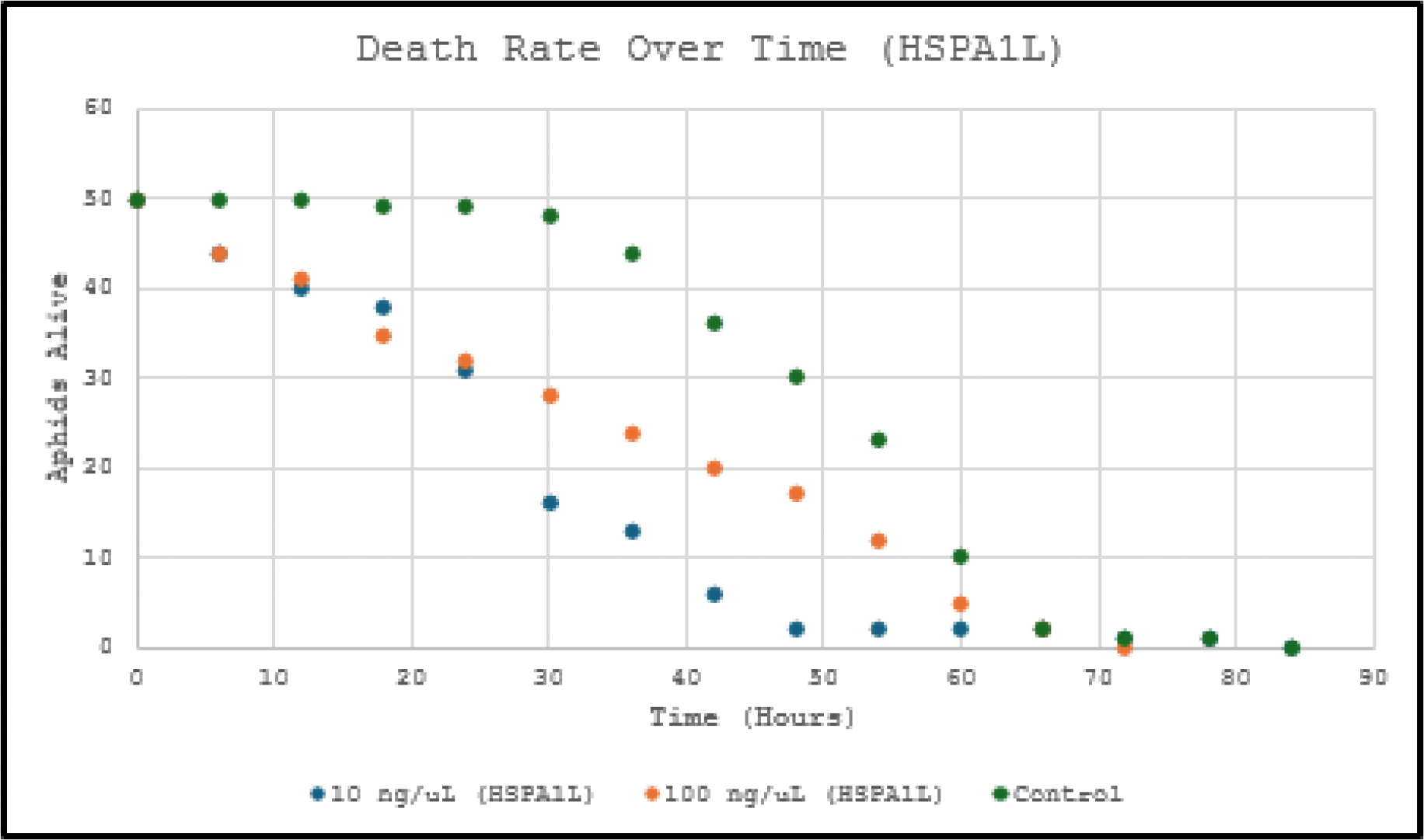
Amount of pea aphids alive (counted every 6 hours) with two concentrations of HSPA1L dsRNA in their diets compared to pea aphids with no dsRNA in their diets. Both concentrations significantly reduced survival vs. control (log-rank p<0.001). Median survival was reduced by ~36–48 h compared to control (~96–108 h).

**Figure 2:**
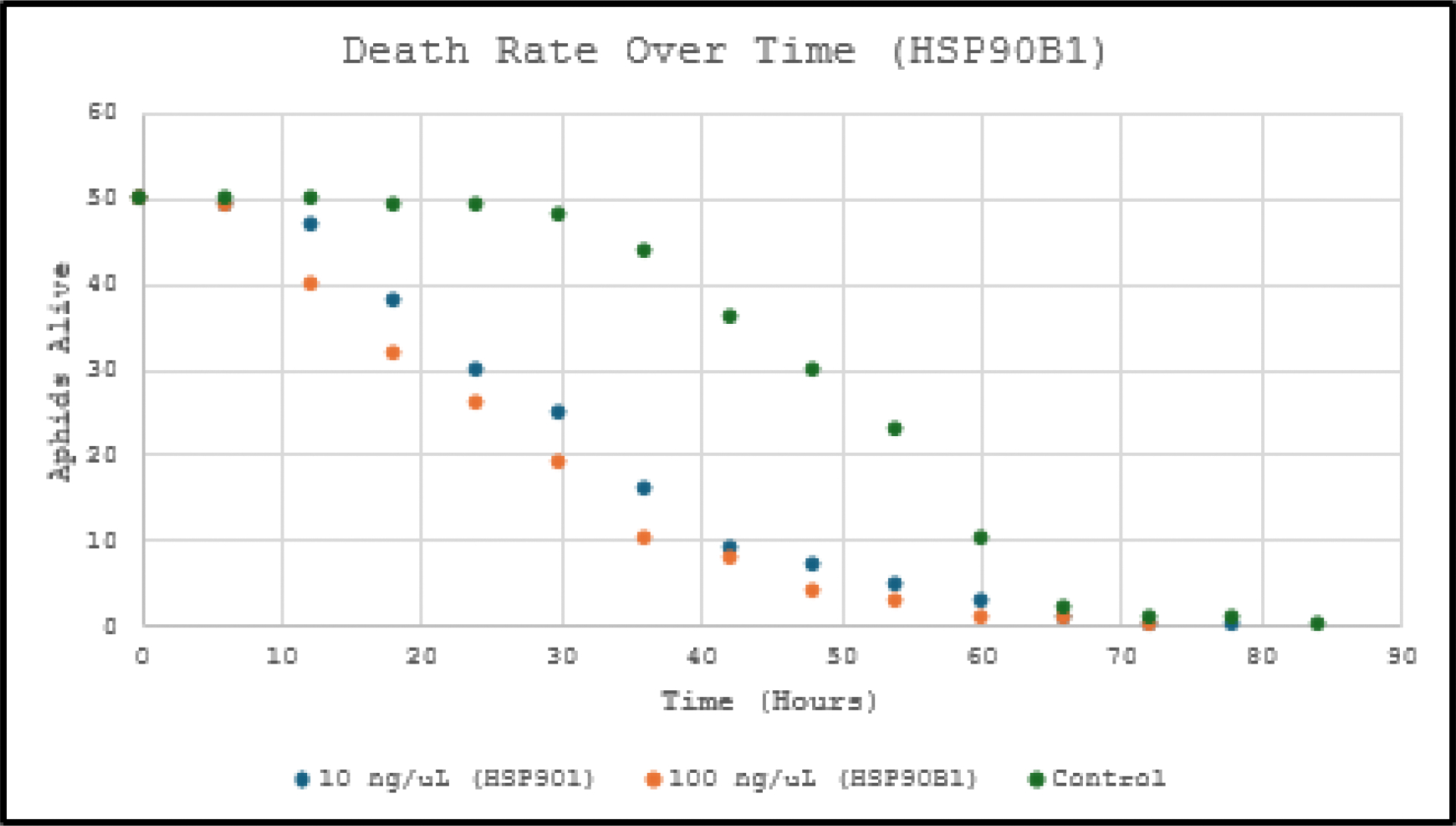
Amount of pea aphids alive (counted every 6 hours) with two concentrations of *HSP90B1* dsRNA in their diets compared to pea aphids with no dsRNA in their diets. Clear dose-dependent effect (log-rank p<0.001 *vs*. control; p<0.05 between 10 ng/μL and 100 ng/μL). The 100 ng/μL group showed the fastest mortality, reducing median survival by ~60–72 h *vs*. control. Both genes significantly decreased overall survival (p<0.001 in all treatment *vs*. control comparisons), confirming apoptosis induction *via UPR* disruption.

Survival data (n=50 aphids per group) were analyzed using Kaplan-Meier survival curves and log-rank (Mantel-Cox) tests (p<0.05 significant) as show in Figures 1 and 2.

## DISCUSSION

dsRNA targeting *HSPA1L* and *HSP90B1* significantly increased mortality (Kaplan-Meier log-rank p<0.001 *vs*. control), supporting the hypothesis that silencing these *UPR*-related heat shock protein genes induces apoptosis *via* proteotoxic stress. *HSP90B1* showed the expected dose-dependent response (p<0.05 between concentrations). *HSPA1L* displayed an unexpected inversion (lower dose slightly more lethal), likely due to variability in ingestion, feeding behavior or encapsulation.

Compared to prior *UPR* gene knockdowns, these genes induced only moderate lethality, suggesting they are not optimal for future multi-gene strategies.

Limitations include uncontrolled timing of initial dsRNA uptake and the diet switch after 48 h (unlike continuous plant-expressed dsRNA).

Overall, the statistically significant results validate *UPR* targeting for species-specific aphid control, while highlighting the need for optimization and stronger target genes.

## CONCLUSION

This study provides clear evidence that oral delivery of dsRNA targeting the heat shock protein genes *HSPA1L* and *HSP90B1* effectively induces apoptosis and reduces survival in *Acyrthosiphon pisum*. Kaplan-Meier survival analysis demonstrated highly significant reductions in aphid lifespan across both tested concentrations (10 ng/μL and 100 ng/μL), with log-rank tests confirming p<0.001 for all treatment groups compared to the untreated control. *HSP90B1* dsRNA exhibited the expected dose-dependent mortality (p<0.05 between concentrations), while *HSPA1L* dsRNA, despite an unexpected dose inversion, still markedly shortened median survival by approximately 36–72 hours relative to controls.

These statistically robust results (p<0.001) support the central hypothesis that disruption of these *UPR*-associated chaperones causes accumulation of misfolded proteins, overwhelms cellular quality control mechanisms, and triggers programmed cell death, leading to elevated aphid mortality. Although the lethality was moderate compared to previously studied *UPR* genes, the consistent and significant survival reduction across replicates validates the approach of targeting heat shock proteins for RNAi-based pest management.

The protocol developed here using BAPC-encapsulated dsRNA delivered through artificial diet-establishes a reliable foundation for species-specific, environmentally safe control of pea aphids. This method avoids broad-spectrum insecticide use and minimizes harm to non-target organisms. Future research will build on these findings by screening additional *UPR* pathway genes for stronger effects on both survival and fecundity, identifying the most potent targets, and testing synergistic multi-gene combinations. Ultimately, the goal is to engineer legume crops that naturally express these aphid-targeted dsRNAs, providing durable, built-in protection against *A. pisum* without reliance on chemical sprays or biological control agents.
